# The effects of residential dual diagnosis treatment on alcohol abuse

**DOI:** 10.15761/JSIN.1000169

**Published:** 2017-07-17

**Authors:** Stephen J Schoenthaler, Kenneth Blum, Lyle Fried, Marlene Oscar-Berman, John Giordano, Edward J. Modestino, Rajendra Badgaiyan

**Affiliations:** 1 Department of Sociology, California State University, Stanislaus, Turlock, CA, USA; 2 Department of Psychiatry & McKnight Brain Institute, University of Florida College of Medicine, Gainesville, FL, USA; 3 Dominion Diagnostics, LLC North Kingstown, RI, USA; 4 National Institute of Holistic Studies, North Miami Beach, FL, USA; 5 Department of Psychiatry, Human Integrated Services Unit University of Vermont Center for Clinical & Translational Science, College of Medicine Burlington, VT, USA; 6 Department of Addiction Research & Therapy, Nupathways, Innsbrook, MO, USA; 7 Department of Psychiatry, Wright State University, Boonshoft School of Medicine, Dayton OH, USA; 8 Department of Psychiatry, University of Southern California, Keck School of Medicine, Los Angeles, CA, USA; 9 Division of Addition Research & Therapy, The Shores Treatment & Recovery Center, Port St. Lucie, FL, USA; 10 Division of Precision Medicine, Geneus Health, LLC, San Antonio, TX, USA; 11 Departments of Psychiatry, Neurology, and Anatomy & Neurobiology, Boston University School of Medicine, and Boston VA Healthcare System, Boston, MA, 02118, USA; 12 Department of Psychology, Curry College, Milton, MA, USA

**Keywords:** Addiction Severity Index (ASI), alcohol abuse, dual diagnosis, relapse, depression, intoxication

## Abstract

This multi-center study of dual diagnosis (DD) programs involved 804 residential patients with co-occurring alcohol and mental health disorders. The Addiction Severity Index was administered at admission and at one, six, and 12 months after discharge. Repeated measures analysis showed the intoxication rate per month stabilized between months six and 12 with 68% still in remission and an 88% mean reduction from baseline (F = 519, p < .005). A comparison between patients with and without weekly relapse produced significant differences in hospitalization (odds ratio 11.3:1; 95% C.I., 5.5 to 23.2). Eight ANCOVAs used mean intoxication days per month after discharge as the outcome variable, pre-admission intoxication days per month as a covariate, and eight variables associated with relapse (e.g. depression) as factors. Patients with these factors at admission did not have significantly higher intoxication rates after discharge than patients without them. This suggests that these DD programs successfully integrated treatment of both disorders and explained their effectiveness. Co-occurring DSM IV mood disorders such as anxiety and depression as well as drug abuse involving opioids or cocaine fell between 66 and 95% at months one, six, and twelve.

## Introduction

The 21st-century increase in dual diagnosis treatment of co-occurring drug and mental health disorders is, in part, a result of the recognition that they typically co-exist and difficulty in achieving long-term remission using treatment-as-usual. Dual diagnosis programs today routinely integrate treatment of both with specific psychosocial interventions [1], medical management [2], motivational interviewing [3], and cognitive behavioral therapy [4,5] using both group and individual counseling based on well-defined treatment principles [6]. The American Society of Addiction Medicine (ASAM) has developed a three-tier taxonomy of addiction-only services (AOS), dual diagnosis capable (DDC), and dual diagnosis enhanced (DDE) services with the difference between the latter two being the capability of integrating treatment of all severities of both disorders [7]. This taxonomy does not imply that AOS or DDC programs are not desirable; some addicts do not have mental health disorders necessitating dual diagnosis treatment and others who might benefit from such treatment do not require DDE services due to low severity. However, disagreement still exists among psychiatry as to whether dually diagnosed patients should receive integrated treatment or referred to addiction-only specialists before commencing mental health treatment [8,9], a question that deserves empirical testing.

Some have indicated [10–12] that there is a lack of well-designed dual diagnosis studies that consider the differences between effectiveness and efficacy. The latter requires randomized controlled trials (RCTs) to determine causation that has high internal validity. The primary limitation of dual diagnosis RCTs is low external validity due to the use of extensive inclusion/exclusion criteria that hinder generalizability to clinical practice. Effectiveness studies require naturalistic, non-experimental designs (NNEDs) which tend to have high external validity due to little or no patient exclusion criteria that allows generalization to patients in clinical settings, but fail to consider internal validity. The primary limitation is that these designs may demonstrate an association, but not causation. McHugo and his colleagues [10] recommend that non-experimental dual diagnosis research should attempt to improve internal validity and recommend six procedures for dual diagnosis research that this study used.

First, “the methods, settings, and interventions of an experiment [should] approximate the real-life situation that is under study.” Second, the study should use interventions that have produced significant results in RCTs. Third; the intervention should utilize residential sites since they produce better outcomes than out-patient services. Fourth, short-term outcomes need to be compared with long-term outcomes since deterioration over time in dual diagnosis research is typical. Fifth, secondary outcomes of interest to patients should be tested to see if they are associated with abstinence. Sixth, moderators that influence response to treatment can be controlled statistically as three-way interactions using analysis of co-variance. These procedures suggested by McHugo and his colleagues [10] make substantial improvements to the internal validity of naturalistic non-experimental designs.

There was methodologic, measurement, and sustainability issues with many of the older studies that caused some to conclude the evidence were not clear that integrated therapies worked better than routine care [13]. Others have concluded [14] that most dual diagnosis patients attain short-term remission of substance use disorders although longer-term relapse is problematic. In a review of dual diagnosis research before the separation of dual diagnosis capable and enhanced classifications, RachBeisel [13] reported that that between 41 and 61% achieved at least short-term remission.

The preceding literature led to three main questions for this study. First, will these three DDE centers collectively produce superior short and one-year outcomes than found in the literature using a repeated measures analysis of alcohol use, intoxication, other illegal drugs, and ASI composite scores? If so, this could provide empirical evidence to support dual diagnosis integrated treatment following a diagnosis of co-occurring disorders. Second, and perhaps most important of all, if the reason that these dual diagnosis programs perform better than sequential treatment is due to their successful treatment of co-occurring mental heal disorders and environmental problems associated with alcohol misuse, that can be empirically tested with these data as follows; among patients who reported psychological, familial, or legal problems at intake, their mean days per month of intoxication during the year after discharge should not be significantly higher than patients who reported no such problems. If these dual diagnosis centers produced excellent outcomes and if there is no association between post-discharge intoxication and these variables, that would be substantial evidence that the reason was due to their sufficiently addressing co-occurring disorders and other problems during integrated dual diagnosis treatment. We found no other study that has ever tested whether dual diagnosis centers can eliminate the association between co-occurring mental health problems at intake and post-discharge relapse. The third question deals with secondary issues of patient concern and public policy, i.e., the utilization of hospital ER visits and admissions due to alcohol and mental health disorders among individuals who become intoxicated weekly when compared to patients who avoid weekly intoxication. We found no other addiction study that has examined this before.

## Material and methods

### Subjects, location, and assessment instruments

The DDE sample came from 1,972 adult patients sequentially admitted to one of three treatment programs in Tennessee or California between 2008 and 2010. Staff in each site administered the Addiction Severity Index [15] and the University of Rhode Island Change Assessment Scale [16] to patients during admission as part of their regular intake process. The sample was reduced to the 1,030 patients who: (a) met DSM clinical criteria for alcohol dependency and reported intoxication during the 30 days prior to admission; and (b) agreed to participate in a study in which they would be asked to re-take the Addiction Severity Index (ASI) periodically after discharge. Attempts were made to interview each former patient at one, six, and 12 months after discharge with 804 of 1,030 (78%) completing at least one of the three post-discharge interviews and 369 completing all three. This resulted in a naturalistic, non-experimental design with high external validity capable of measuring program effectiveness while preserving internal validity using the procedures suggested by McHugo [10].

### Measurement of main outcome variables

Weekly intoxication was defined as any patient who reported weekly intoxication during the previous 30 days or at any time since the previous interview. Mean intoxication days per month was calculated by summing the mean days of intoxication for all reported months and dividing by the number of completed post assessment interviews.

### Ethics

Ethical approval was provided by the institutional review board of Foundations Recovery Network. Informed consent for all patients occurred at admission. Patients were told that if they decided to participate in the study, institutional staff would attempt to locate them at one, six, and twelve months after discharge and repeat the ASI to see how well they are doing. They were told that no service would be withheld if they decided to not participate in the post-discharge research and they could change their mind and withdraw from the study at any time without fear of reprisal.

## Results

There have been minimal differences in patient characteristics at admission to the three dual diagnosis enhanced sites in this study. The sites are similar in days of intoxication, illegal drug use, and co-occurring issues related to mental health, age, race, and gender (Table 1). The primary difference is that the third site is substantially smaller, but still has similar baseline sample characteristics.

The change in alcohol use, intoxication, illegal drug use, and all seven ASI composite scores over three post-tests are given as well as a repeated measures analysis of all ten measures (Table 2). The table consists of data from the 368 patients who completed all assessments, a requirement for repeated measures analysis. The means for each time-period were compared with the means for all 804 participants to examine the effect of missing data (Table 2). Its effect was negligible on the mean changes, i.e., never greater than two percent. The right column contains the results of the repeated measures analyses. With the exception of employment, the means at one, six, and twelve months were always lower than preadmission rates for the other nine variables with each being significant at the .005 level. Furthermore, it is noteworthy that there were no significant differences between the sixth and 12^th^-month assessment for any measure other than employment which continued to improve significantly. The stability between six and 12 months is a new and unexpected finding. Further deterioration is expected with the passage of time due to new relapses exceeding new remissions, but that was not the case among these patients. There were also modest increases in alcohol use, intoxication, and drug use on the sixth-month assessment but they were not followed by increases at the 12^th^-month assessment. The above patterns were consistent among patients who completed all or only some of the post-discharge assessments.

The analysis of the total sample and three subgroups (Table 3): those who reported no intoxication after discharge, those who reported weekly intoxication and those who reported some intoxication that was less than weekly. Reported average intoxication per month during the year after discharge fell from 12,913 to 1,159 (91% less) for the entire sample, an average improvement from 16 to two days of intoxication per month. This was primarily due to 526 (65%) reporting no intoxication during the year after discharge. However, 165 (21%) reported weekly intoxication at some point during the year after discharge, and 120 (15%) reported less than weekly intoxication during this time-period and averaged two days per month.

There was one potential confound (Table 3), i.e., it is possible that former patients who were not reached during one or two of the post-assessments were significantly more likely to have become intoxicated weekly and therefore not available giving a false impression of how few were in this group. This was tested by comparing the proportion who were intoxicated weekly who participated in one, two or all three posttests (chi-square = 0.495, df = 2, p = .78). The proportion was lowest among those who participated all three times, i.e., .21, and highest among those who participated twice at .24 with those who participated once at .22. These results negate the potential limitation of missing data lowering the weekly intoxication rate.

One of the most important issues, theoretically, appears to be an explanation as to why most former dual diagnosis patients maintained remission for one year (Table 4). Previous research has shown that co-occurring problems, such as found in the Problem Severity Index, are associated with significantly higher post-discharge drug use [17]. That was tested two ways on each of eight variables in this table, i.e., with mean intoxication rates and weekly intoxication rates using analysis of variance and analysis of covariance. This table compares patients who had or did not have, at the time of admission, any of these eight measures related to mental health disorders. The presence of or absence or any of these eight measures at admission were not significantly related to higher weekly intoxication or mean days of intoxication after discharge (Table 4). This table suggests that patients who suffered from mental and alcohol disorders, and were treated for both concurrently using dual diagnosis protocols, produced better short and long term alcohol outcomes than sequential treatment because of the effectiveness of the integrated treatment of the co-occurring disorders and other measures that have been historically associated with elevated relapse.

The mean differences in secondary outcomes of interest to patients are given (Tables 5–6) as suggested by McHugo [10]. These tables show that hospitalizations and emergency room visits attributed to alcohol, drugs, and/or mental health problems occurred among one to four percent of patients who never reported becoming intoxicated weekly after discharge (Table 5). However, hospitalizations and emergency room visits attributed to alcohol, drugs, and/or mental health problems occurred among 12 to 31% of patients who reported weekly intoxication. The odds ratios of utilizing various hospital services among those who reported weekly intoxication after discharge varied between 5.8 and 14.3 to 1 depending on the reported problem. The odds of having a related criminal matter increased by 2.1 as well.

We examined hospital service utilization using the dichotomous nominal variable of use or not (Table 5), and the mean number of days in hospitalization and days visiting the ER for problems related to alcohol, drugs, and mental health issues (Table 6). The difference in days of inpatient hospital services, (.71 versus .04) for drugs/alcohol and mental health problems (.58 versus .09) has important economic implications; the saving associated with less hospitalization among those who ceased to become intoxicated weekly may be considerably larger than the cost of dual diagnosis treatment for the entire sample. A similar pattern is found for the number of ER visits due to alcohol and/or drug-related problems as well as visits due to mental health problems. Furthermore, we examined if former patients were still becoming intoxicated weekly, and if so, were they more than twice as likely to have a criminal matter pending, an important secondary concern for patients, clinicians, and the criminal justice system (Table 6).

We also examined at each post-assessment the percent of reduction in days of intoxication among clients in the most common co-occurring DSMIV disorders that included anxiety and mood disorders such as depression, cocaine, opioids and poly-substance abuse. Among clients with co-occurring cocaine abuse disorders, intoxication fell 67%, 77%, and 53% at months 1, 6, and 12. Among clients with co-occurring opioid abuse disorders, intoxication fell 83%, 89%, and 67% at months 1, 6, and 12. Among clients with anxiety disorders, intoxication fell 91%, 89%, and 67% at months 1, 6, and 12. Among clients with other mood disorders, intoxication fell 76%, 67%, and 77% at months 1, 6, and 12. These reductions in alcohol abusing clients with co-occurring polysubstance and mood disorders found in DSMIV answers the question raised in the introduction as to whether simultaneous or sequential treatment works better for specific disorders. In short, alcohol to the point of intoxication fell between 67% and 91% among common co-occurring disorders that are treated in dual diagnosis facilities, results that exceed what is typically reported and suggest concurrent treatment may be superior to consecutive treatment. Secondary support for this conclusion is found in tables 5 and 6 in that both emergency room visits and hospital admission for mental health issues were significantly lower for clients who reported substantial reductions or no intoxication after discharge.

## Discussion

The primary strength of this naturalistic, non-experimental time-series study was the utilization of the methodological and statistical suggestions by McHugo [10] to improve such dual diagnosis research. The repeated measures show that alcohol misuse, illegal drug use, and mental health disorders can remain in remission long term, when defined as one year, for about two-thirds of patients and intoxication per month fell between 88 and 90% depending on whether one includes all patient data or only those who completed all assessments (Tables 3–4). We are unaware of any published experimental or quasi-experimental study that reports this magnitude of success after one year. However, this begs the question of what would happen if this was followed up by a multi-year study in which long-term was defined as two, three, or five years?

Likewise, we are unaware of any other temporal analysis that reported a slight increase in mean alcohol use, intoxication, and illegal drugs at month six followed by no significant improvements or deterioration in these three measures at month 12; that is also a new finding that has ramifications for future research. What looks like “stabilization” is not correct. A few patients relapsing were offset by a few more in remission.

At the sixth month assessment, 33 patients relapsed after complete remission at month one. This suggests the need for further research designed to determine when their increases occurred between month 1 and 6; that could provide insight into its etiology. For example, if remission lasted a few months before relapse, there may be something in the home environment that was not resolved during treatment that could be addressed. If relapse started about a month after discharge, it might reflect unresolved mental or physical health issues during residency.

Using McHugo’s model [10], it follows that weekly empirical measures of mental and physical health status during residency might be able to predict who is likely to be in the group who becomes intoxicated weekly during the year after discharge. This could lead to clinical modifications before discharge for this subset only. As McHugo et al. [10] suggested, if early markers could predict long-term relapse, it should be possible to add a randomized controlled trial component to this smaller group testing various clinical treatments to see what lowers relapse for this smaller subgroup. None of this would have been apparent without a descriptive, naturalistic, non-experimental time-series design to complement previous randomized controlled trials.

The three-way analysis of covariance provides empirical support as to why these dual diagnosis programs performed so well; the eight variables that were associated with relapse in a national study were not so associated in this study. Patients with any one of these eight variables became intoxicated on average one more time per year than patients without the same variables. Presumably, this was because the dual diagnosis sites were quite effective in dealing with these co-occurring mental health and other issues. Two additional limitations remain concerning generalization to all dual diagnosis programs. First, these were dual diagnosis enhanced programs, and it is unknown as to what percent of patients had disorders as severe as to need their enhanced services. Second, all three dual diagnosis sites also incorporate holistic practices such as dialectical behavior therapy [18], acupuncture [19], nutrient dense food/education [20] and yoga since there is growing evidence of effectiveness when used in conjunction with other interventions with high efficacy in these areas [21–23]. The American Psychiatric Association has recently adopted a consistent position [24] namely, that holistic practices may be worthy to use in conjunction with evidence-based practices, but not as an alternative. This is a methodological limitation since it was impossible to determine if these results were due to typical dual diagnosis services alone, or a combination of typical and holistic practices. It remains unknown as to how much these complementary practices altered the results without a comparative study.

Lastly, there is now a need to raise the suggested standard follow-up rate [17] of 70% when the annual relapse rates fall to only about a third of patients over a year, and weekly intoxication is limited to about a fifth. Missing data limit how far results may be generalized. The only solutions are more intensive follow-up procedures that do not violate informed consent built into the design and/or more-costly intensive procedures to do follow-up among a randomly selected sample that could be reached using stratified randomization based on baseline substance use and mental health status to determine who should be sought out among missing former patients.

It is noteworthy that in our follow –up a total of 226 patients did not respond which represents a successful 76% actual responders. While there may be many reasons for these patients to be unresponsive, based on standard literature in terms of substance abuse follow-up the NIMH considers even a 10% response rate as acceptable. Certainly, these lost patients do not necessarily mean that these 226 patients represent failure. More specifically, we looked at the rates of people who were not contacted (n=226) at one or two of the post tests to see if their rates of intoxication before admission or during post-tests were higher than people who completed all three post assignments ; they were not higher at all. Moreover, those who did not participate at any post –test had been intoxicated 15 of 30 days before admission. If failure to participate had been due to more extreme alcoholism, those who were not successfully contacted should have been intoxicated more before admission. In fact, an alternate reason for these patients not to respond would be that they has more serious co-occurring psychiatric disorders, however this too was not the case. It is important also to note that we continued to contact these non-responders and even tried to contact close friends and family as well. It is our opinion that an approximation of failure rate should be the clinical assessment by attending psychiatrists of clients at the time of discharge comparing those receiving routine release and those against medical advice (AMA). Along these lines only 17% of those receiving routine release became intoxicated on follow-up became intoxicated at least one day per month compared to 45% ( N=56) AMA. This three-fold difference demonstrates the importance of professional assessment before charge.

## Summary

Although randomized controlled trials have the highest internal validity and are clearly the best method to measure efficacy, studies like this that are multi-center, multi-modal, naturalistic evaluations have the highest external validity. They offer demonstrable effectiveness and the ability to generalize their findings to clinical practice. The combination of such designs in conjunction with RCTs leads to the most reliable conclusions and the best path forward in substance abuse treatment.

## Figures and Tables

**Table 1:** Patient characteristics at admission to three dual diagnosis enhanced programs

Sites	Combined	La Paloma	Michael’s House	The Canyon
States		Tennessee	California	California
N	804	244	530	30
**Mean N of days used during previous month**				
Alcohol Use	18.5	17.8	20.3	17.5
Intoxication	16.1	15.0	18.8	13.8
Illegal Drug Use	10.6	10.0	12.2	7.0
Multiple Drug Use	8.4	7.7	10.2	4.5
Cannabis	5.0	4.6	6.2	3.8
Sedatives	4.0	3.5	4.6	1.7
Other Opiates	3.7	3.5	1.7	0.4
Cocaine	2.4	2.2	2.9	1.4
Heroin	.82	.83	.89	0
Amphetamines	.81	.66	1.1	1.5
Barbiturates	.42	.26	.84	0
Methamphetamine	.39	.44	.33	0
**Proportion affirmative**				
Awaiting sentencing	.21	.20	.22	.20
Depression	.74	.75	.72	.67
Anxiety or tension	.83	.81	.87	.87
On prescribed medication	.60	.61	.58	.59
**Demographics**				
Age (standard deviation)	37.9 (12.1)	37.9 (11.2)	37.8 (12.4)	40.4 (13.4)
**Race**				
Caucasian	.90	.87	.91	.93
**Gender**				
Male	.57	.55	.58	.47

**Table 2:** Change in alcohol use, intoxication, illegal drug use and ASI composite scores before and after discharge from residential dual diagnosis treatment

N = 368 completing all assessments	One month before admission period 1	One month after discharge period 2	Six months after discharge period 3	12 months after discharge period 4	Mean change from baseline	p < .001 between periods
Illegal drug use days per month	9.5	0.4	1.0	1.2	**91%**	**1 v 2, 3 & 4**
Intoxication days per month	16.34	1.0	2.1	1.8	**90%**	**1 v 2, 3 & 4**
Alcohol use in days per month	18.8	1.4	3.2	3.0	**87%**	**1 v 2, 3 & 4 2 v 3 & 4**
ASI drug composite score	.135	.020	.020	.023	**84%**	**1 v 2, 3 & 4**
ASI alcohol composite score	.618	.109	.125	.120	**81%**	**1 v 2, 3 & 4**
ASI family composite score	.326	.139	.133	.123	**60%**	**1 v 2, 3 & 4**
ASI legal composite score	.121	.075	.051	.030	**50%**	**1 v 2, 3 & 4**
ASI psychiatric composite score	.478	.262	.230	.223	**50%**	**1 v 2, 3 & 4**
ASI medical composite score	.288	.148	.150	.185	**44%**	**1 v 2, 3 & 4**
ASI employment composite score	.388	.464	.384	.368	−**4% @ 1** **+5% @ 4**	**1 v 2, 2 v 3 & 4**

**Table 3:** Change in intoxication after discharge by subsample

Days of intoxication reported per month N = 804	One month before admission	One month after discharge	Six months after discharge	Twelve months after discharge

**Total sample** (88% monthly improvement)				
mean	16.06	1.35	2.38	2.25
std. error	.36	.174	.256	.266
N	804	661	591	525
sum	12,913	891	1,404	1,182

**The subsample with no intoxication after discharge** (100% monthly improvement)				
mean	16.04	0	0	0
std. error	.45	0	0	0
N	526	431	374	332
sum	8,340	0	0	0

**The subsample with weekly intoxication after discharge** (53% monthly improvement)				
mean	17.23	5.85	10.16	8.52
std. error	.72	.72	.91	.94
N	165	131	124	109
sum	2,843	766	1,270	946

**The subsample with some post-intoxication that was less than weekly** (88% monthly improvement)				
mean	14.42	1.18	1.44	2.59
std. error	.97	.30	.20	.60
N	120	106	93	91
sum	1,730	125	134	236

**Table 4:** Lack of significant associations between mental health indices and two post-discharge intoxication measures for 804 dual diagnosis patients using ANCOVA

Characteristics Before Program Admission	Proportion with Weekly Intoxication after	F	p	Mean days of intoxication per month before after		F	P

**Variables associated with mental health disorders**							

**On prescribed meds for psychiatric problems**							
Yes (n = 478)	.17	1.961	.162	16.05	2.42	2.743	.098
No (n =317)	.10			16.02	1.78		

**Major anxiety/tension**							
Yes (n= 663)	.16	5.883	.016	16.15	2.44	0.420	.517
No (n = 136)	.10			15.35	1.02		

**Major depression**							
Yes (n = 592)	.16	0.277	.599	16.47	2.43	0.006	.793
No (n = 207)	.14			14.72	1.54		

**Violence control difficult**							
Yes (n = 162)	.14	2.425	.120	16.40	2.34	.179	.186
No (n = 636)	.15			15.91	2.17		

**Concentration or Memory difficulties**							
Yes (n = 412)	.16	.475	.491	16.30	2.08	.430	.512
No (n = 387)	.14			15.76	2.32		

**Hallucinations**							
Yes (n = 54)	.14	.813	.367	14.61	1.83	.234	.629
No (n = 745)	.15			16.15	2.23		

**Serious suicide thoughts**							
Yes (n = 147)	.11	1.382	.240	16.00	2.15	.029	.865
No (n = 653)	.16			16.04	2.21		

**Suicide attempts**							
Yes (n = 47)	.15	.004	.953	14.94	1.12	1.75	.186
No (n = 752)	.15			16.11	2.25		

**Totals** N =804	.15			16.06	2.24		

**Table 5:** Odds ratios of secondary results of high patient interest after discharge

Post-discharge Addiction Severity Index secondary measures of high interest to patients		Post-discharge weekly intoxication since last interview		Chi square _____ p	Odds ratio _____ 95% CI
		No	Yes		

Hospitalized alcohol and/or drug related Problems?	yes due to	11 (2%)	29 (17%)	64.47 _____ < .001	11.3 _____ 5.5 to 23.2
	no	606 (98%)	141 (83%)		

Hospitalized due to mental health related problems?	yes	8 (1%)	16 (11%)	34.38 _____ <.001	9.0 _____ 3.8 to 21.4
	no	601 (99%)	134 (89%)		

ER visit due to alcohol and/or drug related problems?	yes	16 (3%)	40 (24%)	88.88 _____ < .001	11.6 _____ 6.3 to 21.4
	no	600 (97%)	129 (76%)		

ER visit due to mental health related problems?	yes	8 (1%)	16 (11%)	34.38 _____ <.001	9.0 _____ 3.8 to 21.4
	no	601 (99%)	134 (89%)		

Have a related pending criminal matter?	yes	43 (8%)	24 (15%)	6.67 _____ = .014	2.0 _____ 1.2 to 3.4
	no	491 (92%)	137 (85%)		

**Table 6:** Mean differences in secondary outcomes of interest to patients with important public policy implications

Secondary outcomes		N	Mean days or incidents	Std. error	t	p

Hospitalized due to alcohol drug related problems?	yes and/or	170	.71	.186	6.65	<.001
	no	617	.04	.014		

Hospitalized due to mental health related problems?	yes	166	.58	.253	3.02	=.003
	no	615	.09	.050		

ER visit due to alcohol and/or drug related problems?	yes	169	.46	.088	8.19	<.001
	no	616	.04	.011		

ER visit due to mental health related problems?	yes	169	.22	.050	2.24	=.025
	no	617	.07	.042		

Have a related pending criminal matter?	yes	161	.27	.056	3.13	=.002
	no	534	.12	.020		
